# Identification of Functional Bioprocess Model for Recombinant *E. Coli* Cultivation Process

**DOI:** 10.3390/e21121221

**Published:** 2019-12-14

**Authors:** Renaldas Urniezius, Arnas Survyla

**Affiliations:** Department of Automation, Kaunas University of Technology, Kaunas LT-51367, Lithuania; arnas.survyla@ktu.lt

**Keywords:** gray box, relative entropy, microbial cultivation, numerical convex optimization, parameter estimation, stoichiometry

## Abstract

The purpose of this study is to introduce an improved Luedeking–Piret model that represents a structurally simple biomass concentration approach. The developed routine provides acceptable accuracy when fitting experimental data that incorporate the target protein concentration of *Escherichia coli* culture BL21 (DE3) pET28a in fed-batch processes. This paper presents system identification, biomass, and product parameter fitting routines, starting from their roots of origin to the entropy-related development, characterized by robustness and simplicity. A single tuning coefficient allows for the selection of an optimization criterion that serves equally well for higher and lower biomass concentrations. The idea of the paper is to demonstrate that the use of fundamental knowledge can make the general model more common for technological use compared to a sophisticated artificial neural network. Experimental validation of the proposed model involved data analysis of six cultivation experiments compared to 19 experiments used for model fitting and parameter estimation.

## 1. Introduction

Biotechnology plants seek to increase the productivity and controllability of cell cultivations. In order to achieve those two quality conditions, they need a trustworthy data collection system that provides mandatory variables in real time to smoothly control processes and achieve the required productivity. A system like this would require compatible equipment that might restrict access due to financial concerns, because it is not compatible with the chosen system or may lack functionality. However, it is worth replacing sophisticated equipment with soft sensors that estimate the desired non-observable parameters from the measured data collected [1,2].

In previous works [3], the biomass estimation model relied on stoichiometry, where biomass maintenance eventually proved to be the third-order polynomial term. The biomass maintenance term consists of oxygen consumption, not only for maintenance, but also for product synthesis [4]. This study suggests fundamental knowledge based on the Luedeking–Piret model [5], in which the infrastructure of the maintenance term consists of both actual biomass maintenance and target protein production. In this case, the proposed model is clearer and can achieve greater accuracy. The main aim of this paper is to estimate the Luedeking–Piret model parameters in the offline mode using product information. Simultaneously, this paper provides an alternative way to fit a protein production model and analyze the parameters of the model based on offline data. To date, few studies and publications have estimated the state of target proteins using a soft-sensor approach. The majority of published works estimating target protein productivity and biomass concentration use an artificial neural network (ANN) approach. The novelty of this study is that it involves the fundamental knowledge of incorporating target protein synthesis into a product and biomass concentration model. This results in a rational parametric model that can serve as an alternative approach to ANN. Parameters of the proposed model of estimation have practical significance; therefore, approach-related artifacts are less expected and their elimination is manually controllable during the development of industrial processes.

Section 2: Materials and Methods describe the materials, strains, and bioreactor system operating conditions. Section 3: Basis of Biomass and Product Model Fitting reviews the idea and basis of this study. Section 4: System Identification and Parameter Estimation presents the derivation of a known method for fitting to target protein and biomass concentration models. It also lays out a general formula for oxygen consumption according to the stoichiometric coefficients of biomass, which is relevant to the specific culture of Escherichia coli. Section 5: Experimental Validation provides results from experimental data supporting the validity of the improved Luedeking–Piret model and an offline estimation of target protein and biomass concentrations. Section 6: Conclusions discusses the results and concludes the final statements of this study.

## 2. Materials and Methods

### 2.1. Cell Strains

In this work, *E. coli* BL21 (DE3) pET28a (Novagen) served as the test object in all experiments [4] in order to validate biomass and protein model fitting. The product of *E. coli* BL21 (DE3) appeared in two forms: Active soluble and insoluble forms, which were formed as inclusion bodies. In this study, the target product was insoluble protein, inclusion bodies. The protein’s expression was under the control of the T7 promoter after induction with 1 mM isopropyl-D-1-thiogalactopyranoside (IPTG).

### 2.2. Medium and Culture Conditions

Experimental data [6,7,8] served as the basis for analysis in this study. Genetically modified *E. coli* BL21 (DE3) pET28a strain was cultivated in a B. Braun 10 L bioreactor. Due to confidentiality restrictions, the authors of Reference [6] claimed that the organism expressed commercial protein, and no specific details are available on the target recombinant protein. The initial medium volume at inoculation was 5 L. The cultivation medium contained mineral salt medium, consisted of Na_2_SO_4_, 2.0 g/L; (NH_4_)_2_SO_4_, 2.46 g/L; NH_4_Cl, 0.5 g/L; K_2_HPO_4_, 14.6 g/L; NaH_2_PO_4_ × H_2_O, 3.6 g/L; (NH_4_)_2_–H–citrate 1.0 g/L; MgSO_4_ × 7H_2_O, 1.2 g/L; trace element solution, 2 mL/L; thiamine, 0.1 g/L; and kanamycin, 0.1 g/L [6]. Cultivation experiments took place in fed-batch mode with zero glucose concentration in the bioreactor at the inoculation time. Pumping of the feed solution containing glucose and mineral salts in the same composition as the starting medium started after inoculation in the bioreactor [7]. During all experiments, after inoculation, the initial biomass inside the bioreactor was 0.25 g/L of dry cell weight (DCW). At the beginning of cultivation, the feed rate of the substrate was set very low, approximately 11–15 g/h, and used substrate solution with as low as 300 g/kg glucose concentration to avoid overdose, which resulted in substrate inhibition or a different metabolic pathway. At ~4 g/L biomass concentration in medium, feed solution of 600 g/kg replaced the one with 300 g/kg glucose concentration [8]. The temperature set point in the bioreactor was set at 35 °C. The induction time was 10 h since inoculation. Tracking of off-gassing from the bioreactor was done online, and a paramagnetic oxygen sensor (Maihak Oxor 610) operated for O_2_ concentration observation. An Ingold DO probe (Mettler Toledo) measured dissolved oxygen tension (DOT) values. The DOT set point was set to 25% of saturation [9]. pH was measured with an Ingold pH probe (Mettler Toledo) and kept at 7.0 using a PID controller [10]. After the action of cell disruption, separation of the soluble fraction, and solubilization of inclusion bodies, SDS-PAGE electrophoresis helped to determine the amount of the target protein. The method for measuring protein concentration (g/L) consisted of several preparation steps. Initially, 200 g of wet biomass was dissolved in 1 mL of solution and mixed for 30 min. After that SDS-PAGE, (sodium dodecyl sulfate polyacrylamide gel) electrophoresis was performed on 200 µL of the suspension sample to measure the amount of total protein concentration. The remainder of the suspension was mixed with SDS (sodium dodecyl sulfate) buffer to dissolve all proteins and centrifuged for 15 min at 4 °C with 20,000 G force. After centrifugation, SDS-PAGE electrophoresis was used to determine the soluble protein concentration in a 200 µL sample. The remaining supernatant discarded and replaced with 1 mL of water, then mixed and centrifuged. After decanting the supernatant, 1 mL of solubilization buffer (8M urea; 50 mM, pH 8.0 Tris base) was added and mixed for approximately 12 h. The final step after mixing was the measuring of insoluble protein (inclusion bodies) concentration with SDS-PAGE electrophoresis.

## 3. Basis of Biomass and Product Model Fitting

A previous study [3] showed that the development of a biomass concentration estimator required data that was linked to the biomass growth rate. Oxygen uptake rate (OUR) was the main characteristic variable that provided information about the biosynthesis phenomenon [7,11,12]. To enforce soft sensors [13,14], OUR must have been an online measurement coming from devices that registered not only mass airflow, but also O_2_ concentration in the off-gas [15]. This study proposes biomass concentration and protein model fitting based on a mass balance equation. For fed batch cultivations, such a model originates from the Luedeking–Piret model. The mass balance model represents the relationship between oxygen uptake rate (OUR) and biomass growth characteristics [5]:

(1) $$\mathrm{OUR}\left(t\right)=\alpha \cdot X{}^{\prime }\left(t\right)+\beta \cdot X\left(t\right)$$

In Equation (1), X is dry biomass concentration (g/L), t is duration time since inoculation (h) and stoichiometric coefficients α and β represent cell metabolism of oxygen consumption, where α describes the cell’s oxygen consumption yield for biomass growth ($\alpha \equiv Y_{o_{2}/X},\left[\;g\left(O_{2}\right)/g\left(X\right)\right]$) and β describes the cell’s oxygen consumption for maintenance ($\beta \equiv m_{O_{2}/X},\left[g\left(O_{2}\right)/g\left(X\right)/h\right]$) [16,17,18]. Çalik [19], studying the effects of pH on benzaldehyde lyase production by *Escherichia coli*, and Kocabaş [20], studying l-tryptophan production, clarified that oxygen consumption consisted of three parts: Cell growth, maintenance, and byproduct formation. In order to enable model fitting of protein and biomass concentration, this study suggests modifying the Luedeking–Piret model in Equation (1) by adding parameter γ, which represents the oxygen consumption yield for protein synthesis rate $\;P\;{}^{\prime }\left(t\right)$ ($\gamma \equiv Y_{o_{2}/p}$) [4,21]:

(2) $$\mathrm{OUR}\left(t\right)=\alpha \cdot \;X\;{}^{\prime }\left(t\right)+\beta \cdot X\left(t\right)+\gamma \cdot \;P\;{}^{\prime }\left(t\right),$$

where the last term represents the oxygen update rate for product formation.

## 4. System Identification and Parameter Estimation

### 4.1. Stoichiometric Parameter Estimation

In a previous study [3], there was an assumption that stoichiometric parameter β, the oxygen maintenance term, was not a process constant, and one explanation was that it embraced the target protein P production:

(3) $$\mathrm{OUR}\left(t\right)=\alpha \cdot \;X\;{}^{\prime }\left(t\right)+\beta \left(X\right)\cdot X\left(t\right),$$

where the β function had the form

(4) $$\beta \left(X\right)\equiv \beta \left(X\left(t\right)\right)=k_{\beta 2}\cdot X^{2}\left(t\right)+k_{\beta 1}\cdot X\left(t\right)+k_{\beta 0}.$$

**Figure 1:**
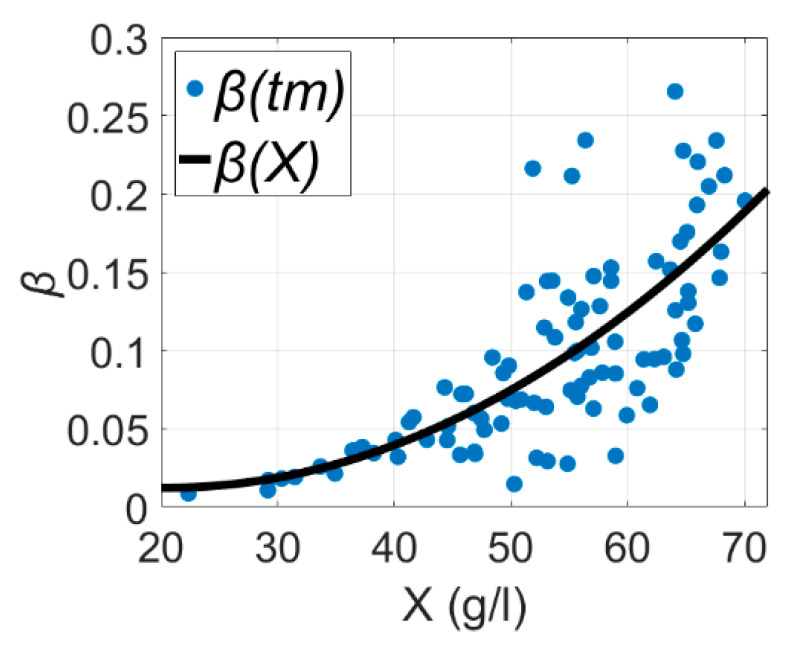
Dependence of oxygen consumption for maintenance on biomass concentration of *E. coli* estimated as a function of biomass and observed at discrete time $t_{m}$, taken from Reference [3].

Equation (3) gives acceptable results, but is highly uncertainty for the β term [3], which can be seen in Figure 1, where β(X) is the maintenance coefficient as a function of biomass concentration and β(tm) is the maintenance coefficient observed at discrete time $t_{m}$ and is associated with biomass X at time $t_{m}$. Graph data for Figure 1 originated from Reference [3].

In order to refine the model to a simpler and more versatile one, an additional parameter γ(X) extends the parsimonious model [22] to the shape of Equation (5):

(5) $$\mathrm{OUR}\left(t\right)=\alpha \cdot \;X\;{}^{\prime }\left(t\right)+\beta \left(X\right)\cdot X\left(t\right)+\gamma \left(X\right)\cdot \;P\;{}^{\prime }\left(t\right).$$

This represents the main novelty of this study, protein production yield γ [4,21], which is assumed to be a function of biomass concentration X in a gray box model of Equation (3) [6,22]. The motivation of Equation (5) is that, through a convex programming procedure, the parameters with higher statistical significance overcome those with lower significance by leaving their entries populated with zero values. Babaeipour et al. [23] showed that protein productivity depends on IPTG and biomass concentrations at the induction time. In previous research [6], experiments had the same 1 mM amount of isopropyl-D-1-thiogalactopyranoside (IPTG). However, the biomass concentration at the induction time in each cultivation process was different. We found that it had a significant impact on the biomass model fitting. Our analysis showed that the product formation parameter γ(X) is a function of biomass concentration at induction time [24]:

(6) $$\;\gamma \left(X\right)=k_{\gamma }\cdot \left(X\left(t\right)-X_{\mathrm{ind}}\right),$$

where X_ind_ is biomass concentration at induction time and $k_{\gamma }$ is the product synthesis yield, which is assumed to be constant. In summary, the full gray box model of the estimator has the form:

(7) $$\mathrm{OUR}\left(t\right)=\alpha \cdot \;X\;{}^{\prime }\left(t\right)+k_{\gamma }\cdot \left(X\left(t\right)-X_{\mathrm{ind}}\right)\cdot \;P\;{}^{\prime }\left(t\right)+\;\left(k_{\beta 2}\cdot X^{2}\left(t\right)+k_{\beta 1}\cdot X\left(t\right)+k_{\beta 0}\right)\cdot X\left(t\right).$$

In electrical systems, disturbances and interferences are inevitable, and the model’s parameters and estimated values are distorted [11]. Urniezius et al. [3] and Schaepe et al. [13] showed that cumulative signals had less disturbance and an improved signal-to-noise ratio (SNR). In order for the original signal to be cumulative, this study employs an integral approach, which is a good noise filter [25]. After integration, the improved Luedeking–Piret model in Equation (7) becomes more resistant to state variable noise [26]:

(8) $$\int_{t_{0}}^{t}\mathrm{OUR}\left(t^{*}\right)dt^{*}=\alpha \cdot \int_{t_{0}}^{t}\;X\;{}^{\prime }\left(t^{*}\right)dt^{*}+k_{\gamma }\cdot \int_{t_{0}}^{t}\left(X\left(t^{*}\right)-X_{\mathrm{ind}}\right)\cdot P{}^{\prime }\left(t^{*}\right)dt^{*}+\;\int_{t_{0}}^{t}\left(k_{\beta 2}\cdot X^{2}\left(t^{*}\right)+k_{\beta 1}\cdot X\left(t^{*}\right)+k_{\beta 0}\right)\cdot X\left(t^{*}\right)dt^{*}.$$

After model analysis and calculations, the obtained results show that the stoichiometric parameter β(X), the oxygen maintenance term for biomass concentration, is extremely low in comparison to other stoichiometric parameters during the whole cultivation process. The convex estimation of coefficients $k_{\beta 2},{\;k}_{\beta 1},{\;k}_{\beta 0}$, manifested in this study, shows that all of those coefficients obtain zero values in this parsimonious model. The phenomenon where the biomass maintenance factor is absent from the growth process can be explained by the fact that the biomass concentration at the induction moment is relatively low (around 30 g/L) and the biomass maintenance term is negligible in this specific situation. A previous study [3] presented biomass maintenance model fitting procedures; therefore, Equation (8) considers only two terms of oxygen consumption:

(9) $$\int_{t_{0}}^{t}\mathrm{OUR}\left(t^{*}\right)dt^{*}=\alpha \cdot \int_{t_{0}}^{t}X{}^{\prime }\left(t^{*}\right)dt^{*}+k_{\gamma }\cdot \int_{t_{0}}^{t}\left(X\left(t^{*}\right)-X_{\mathrm{ind}}\right)\cdot P{}^{\prime }\left(t^{*}\right)dt^{*}.$$

Integration with parts [27] of the last term in Equation (9) enables model fitting of biomass concentration. This helps to remove the protein production rate containing considerable uncertainty:

(10) $$\int_{t_{0}}^{t}\mathrm{OUR}\left(t^{*}\right)dt^{*}=\alpha \cdot \left(X\left(t\right)-X\left(t_{0}\right)\right)+\;\;k_{\gamma }\cdot \left(P\left(t\right)\cdot \left(X\left(t\right)-X_{\mathrm{ind}}\right)-\int_{t_{0}}^{t}\left(X\left(t^{*}\right)-X_{\mathrm{ind}}\right){}^{\prime }\cdot P\left(t^{*}\right)dt^{*}\right),$$

The differential of current biomass concentration minus biomass concentration at induction time simplifies to ${\left(X\left(t^{*}\right)-X_{\mathrm{ind}}\right)}^{\prime }=\frac{d\left(X\left(t^{*}\right)-X_{\mathrm{ind}}\right)}{dt^{*}}=\frac{dX\left(t^{*}\right)}{dt^{*}}$. Therefore,

(11) $$\int_{t_{0}}^{t}\left(X\left(t^{*}\right)-X_{\mathrm{ind}}\right){}^{\prime }\cdot P\left(t^{*}\right)dt^{*}=\int_{t_{0}}^{t}\frac{dX\left(t^{*}\right)}{dt^{*}}\cdot P\left(t^{*}\right)dt^{*}=\;\int_{t_{0}}^{t}P\left(t^{*}\right)dX\left(t^{*}\right)\approx \sum_{l=1}^{m}\left(X_{l}-X_{l-1}\right)\cdot P_{l},$$

where the last integral of Equation (11) represents the left-hand Riemann sum [28], when the time’s t sample has an index of m. Discrete DCW samples define variable $X_{m}\equiv X\left(t\right)$, where $m\in \left[1,n_{m}\right]$; $n_{m}\;$ is the total number (hours) of offline sampling intervals and $X_{0}\equiv X\left(t_{0}\right)$ is an initial biomass concentration after inoculation in the bioreactor. Introducing ${\mathrm{cOUR}}_{m}\equiv \int_{t_{0}}^{t}\mathrm{OUR}\left(t^{*}\right)\;dt^{*}$ and Equation (11) into Equation (10) yields:

(12) $${\mathrm{cOUR}}_{m}=\alpha \cdot \left(X_{m}-X_{0}\right)+k_{\gamma }\cdot \left(P_{m}\cdot \left(X_{m}-X_{\mathrm{ind}}\right)-\sum_{l=1}^{m}\left(X_{l}-X_{l-1}\right)\cdot P_{l}\right).$$

The final formula for offline model fitting of biomass concentration is as follows:

(13) $$X_{m}=\frac{{\mathrm{cOUR}}_{m}+\alpha \cdot X_{0}+k_{\gamma }\cdot P_{m}\cdot X_{\mathrm{ind}}+k_{\gamma }\cdot \sum_{l=1}^{m}\left(X_{l}-X_{l-1}\right)\cdot P_{l}}{\alpha +P_{m}\cdot k_{\gamma }}.$$

Equation (13) also represents the prediction value of the proposed model, i.e., it serves as the constraint over the probabilistic mean $\langle X_{m}\rangle$.

### 4.2. Procedure for Offline Analysis of Stoichiometry Parameters

Fitting the biomass concentration parameters to the gray box model means that the analysis of offline bioprocess data evaluates the stoichiometric parameters of the cell strain. Equation (13) shows that the essential data must consist of dry cell weight (DCW) or an optical density (OD) value (o.u.), which is converted to DCW by multiplying it by a factor of 0.4 g/L/o.u. [29], cumulative oxygen uptake rate (cOUR), and insoluble target protein values. However, the time duration of the process since inoculation is not required during this gray box model fitting procedure.

The model for fitting parameter values is a gray box model, because collected experimental data are combined with fundamental knowledge about bioprocesses [30]. The posterior distribution for the m-th offline sample is:

(14) $$P_{\mathrm{posterior}}\left(X_{m}\right)\sim N\left(\langle X_{m}\rangle ,\sigma_{\langle X\rangle }^{2}\right),$$

where every sampled prediction m has a constant variance $\sigma_{\langle X\rangle }^{2}$. Prior distribution also has the form of Gaussian distribution [31,32]:

(15) $$P_{\mathrm{likelihood}}\left(X_{m}\right)\sim N\left(X_{m}^{y},\sigma_{X,m}^{2}\right),$$

where $X_{m}^{y}$ is the mth observation value of the biomass concentration and its individual variance is $\sigma_{X,m}^{2}$. Integration of relative entropy [31] yields:

(16) $$\begin{array}{ll} S_{m}\left(P_{\mathrm{posterior}},P_{\mathrm{likelihood}}\right) & =-\int_{-\infty }^{\infty }P_{\mathrm{posterior}}\left(X_{m}\right)\cdot \ln\frac{P_{\mathrm{posterior}}\left(X_{m}\right)}{P_{\mathrm{likelihood}}\left(X_{m}\right)}dX_{m}\\ & =-\frac{{\left(\langle X_{m}\rangle -X_{m}^{y}\right)}^{2}}{2\cdot \sigma_{X,m}^{2}}+c, \end{array}$$

where a further procedure neglects coefficient c. In a previous study [31], the uncertainty of prior distribution was set as equal to the squared observed value. However, Reference [3] showed that there are trade-offs between the least squares approach and the squared mean absolute percentage error (MAPE) criterion. A separate tuning coefficient $K_{\exp}$ [3] is required to adjust uncertainty:

(17) $$\sigma_{X,m}^{2}\sim \frac{X_{m}^{2}}{1-K_{\exp}+X_{m}^{2}\cdot K_{\exp}},$$

which yields the sum of two criteria after insertion into Equation (16)

(18) $$\begin{array}{ll} S_{m}\left(P_{\mathrm{posterior}},P_{\mathrm{likelihood}}\right) & =-\frac{{\left(\langle X_{m}\rangle -X_{m}^{y}\right)}^{2}}{2\cdot \frac{X_{m}^{2}}{1-K_{\exp}+X_{m}^{2}\cdot K_{\exp}}},\\ & =-\frac{{\left(\langle X_{m}\rangle -X_{m}^{y}\right)}^{2}\cdot \left(1-K_{\exp}\right)}{2\cdot X_{m}^{2}}-\frac{{\left(\langle X_{m}\rangle -X_{m}^{y}\right)}^{2}\cdot K_{\exp}}{2}. \end{array}$$

The tuning coefficient $K_{\exp}$ $\left(0\leq K_{\exp}\leq 1\right)$ with a value of 1 recreates the least squares approach, which has a higher penalty for bigger criterion values. Meanwhile, the value of zero results in the squared MAPE criterion [31], which restricts estimation errors to smaller overall criterion values. Such criteria showed acceptable practical benefits in a generic case of a biomass model fitting procedure. As a result, the least squares method is combined with the squared MAPE to apply the advantages of both criteria and top overcome their disadvantages, where $K_{\exp}$ is an empirical “weight” coefficient between the two additive terms of the optimization criterion.

### 4.3. Model of Product Model Fitting

Product evaluation technology is a complex soft sensor and is important for the biotechnology industry, demonstrating process efficiency and saving time in protein measurements [9]. In this study, the basic idea of the protein model fitting comes from Levisauskas’ research [33], claiming that relative protein synthesis is a function of the specific biomass growth rate:

(19) $$\frac{dP_{x}}{dx}=q_{\mathrm{px}}\left(\mu ,{\;P}_{x}\right),$$

where $q_{\mathrm{px}}$ is a specific protein accumulation rate (U/g/h), µ is a specific biomass growth rate (1/h), and $P_{x}$ is specific protein activity (U/g), where protein concentration is divided by biomass concentration, $P_{x}\left(t\right)=P\left(t\right)/X\left(t\right)$ [33]. Data analysis and studies have shown that production synthesis is linearly dependent on the specific growth rate (SGR) of the biomass and the product concentration acts as an inhibitor of product synthesis [34]:

(20) $$\frac{dP_{x}}{dx}=q_{\mathrm{px}}\left(\mu ,{\;P}_{x}\right)=P_{\max}\left(\mu ,\;X\right)-k_{t}\cdot P_{X}\left(t\right).$$

In Equation (16), coefficient $k_{t}$ is a corresponding time constant that is assumed to be a form of the self-inhibition effect [35]. $P_{\max}$ is a maximal specific product concentration value, which is asymptotically dependent on μ. The specific biomass growth rate and biomass concentration determine the maximum specific product concentration term [36], which represents the maximum possible protein concentration in the current process state:

(21) $${\;P}_{\max}\left(\mu ,\;X\right)=\mu \left(t\right)\cdot \left(k_{m0}+k_{m1}\cdot \left(X\left(t\right)-X_{\mathrm{ind}}\right)\right),$$

where $k_{m0}$ and $k_{m1}$ are empirical coefficients proposed by this study, $k_{m0}$ relates to SGR and protein synthesis, and $k_{m1}$ links the biomass concentration at the induction time and productivity [23]. Equation (21) is only valid after induction and biomass concentration at induction time is a prerequisite. Before IPTG injection into the bioreactor, coefficient $k_{m1}$ is equal to zero and the maximum specific product concentration term becomes:

(22) $$P_{\max}\left(\mu ,\;X\right)=\mu \left(t\right)\cdot k_{m0}.$$

The protein model fitting is comparable to the gray box model and the biomass concentration model. Prior to performing the coefficient evaluation, the gray box model, represented by Equation (20), integrates to:

(23) $$P_{X}\left(t\right)=\overset{t}{\underset{t_{0}}{\int }}P_{\max}\left(t^{*}\right)dt^{*}-k_{t}\cdot \int_{t_{0}}^{t}P_{X}\left(t^{*}\right)dt^{*}$$

The integrals of Equation (23) are expressed as the left-hand Riemann sum [28], i.e., $\int_{t_{0}}^{t}P_{\max}\left(t^{*}\right)dt^{*}\approx \sum_{j=1}^{m}P_{\max,j}\cdot ∆t_{j,j-1};\;\int_{t_{0}}^{t}P_{X}\left(t^{*}\right)dt^{*}\approx P_{X,m}\cdot ∆t_{m,\;m-1}+\sum_{j=1}^{m-1}P_{X,j}\cdot ∆t_{j,j-1}$; when time’s t sample is indexed by m, discrete protein values define the variable${\;P}_{X,m}\equiv P_{X}\left(t\right),$ where $m\in \left[1,n_{m}\right]$. The final formula of protein model fitting is as follows:

(24) $$P_{m}=\;\frac{\left(\sum_{j=i}^{m}P_{\max,j}\cdot ∆t_{j,j-1}-k_{t}\cdot \sum_{j=1}^{m-1}P_{x,j}\cdot ∆t_{j,j-1}\right)\cdot X_{m}}{1+∆t_{m,m-1}\cdot k_{t}}.$$

Model fitting uses Equation (24) for a prediction value $\langle P_{m}\rangle \;$and observed product concentrations $P_{m}^{y}$ inside convex optimization.

### 4.4. Identification of E. Coli Parameters by Convex Optimization

**Figure 2:**
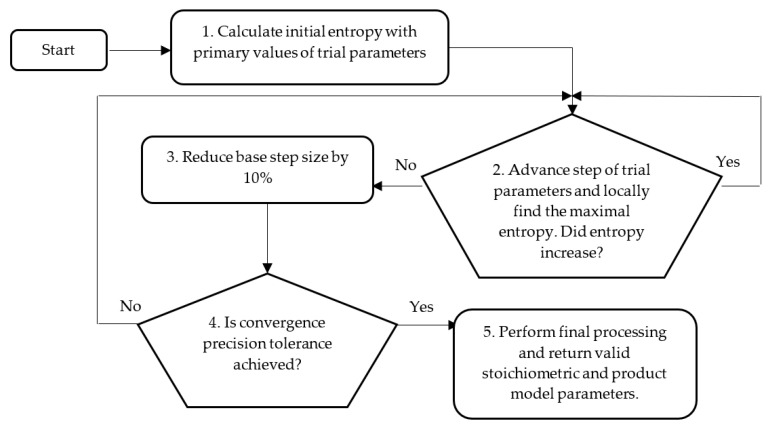
Workflow of structural scheme for convex optimization method identifying stoichiometric and product model fitting parameters.

The process of identifying *E. coli* BL21 (DE3) pET28a strain’s stoichiometric parameters and protein model fitting coefficients is based on the convex optimization method and the maximization of entropy [31,37]. Figure 2 depicts the workflow of the optimization procedure.

Convex optimization uses the maximization of entropy as an indicator of local extremum detections [38]. Equation (18) helps with identification of stoichiometry parameters and Equation (25) does the same for the product model fitting:

(25) $$S_{P,m}=-\frac{{\left(\langle P_{m}\rangle -P_{m}^{y}\right)}^{2}\cdot \left(1-K_{\exp}\right)}{2\cdot X_{m}^{2}}-\frac{{\left(\langle P_{m}\rangle -P_{m}^{y}\right)}^{2}\cdot K_{\exp}}{2}$$

## 5. Experimental Validation

For the comparison of results, the mean absolute error (MAE) and mean absolute percentage error (MAPE) are operated as evaluation criteria. The definition of MAE is [39]:

(26) $$\mathrm{MAE}=\frac{\sum_{i=1}^{n}\left|{\hat{y}}_{i}-y_{i}\right|}{n},$$

where n is the number of data counts, ${\hat{y}}_{i}$ is the estimation result, and $y_{i}$ is the observed value from the cultivation process. MAPE has the expression [40]:

(27) $$\mathrm{MAPE}=\frac{100\;\%}{n}\overset{n}{\underset{i=1}{\sum }}\left|\frac{{\hat{y}}_{i}-y_{i}}{y_{i}}\right|.$$

Root mean square error (RMSE) represents the square root of the residuals of the differences between predicted and observed values. The formula is as follows [39]:

(28) $$\mathrm{RMSE}=\sqrt{\frac{\sum_{i=1}^{n}{\left({\hat{y}}_{i}-y_{i}\right)}^{2}}{n}}.$$

The experimental data of the fed-batch cultivation process of *Escherichia coli* were taken from Reference [6]. In order to test and validate the proposed models of this paper, data from 19 cultivation experiments were used in the system identification analysis. The start of this research included investigating a suitable expression describing stoichiometry parameters in biomass model fitting. Multiple tests employed various formulations, including previous assumptions on polynomial maintenance [3]. The purpose was to indicate the most suitable formula that describes cell stoichiometry. Table 1 describes the best-achieved coefficient values for the fitted model.

The MAE and MAPE values show the average from 19 experiments. The results of protein model fitting Equation (24) are presented in Table 2.

Table 2 presents the model parameters that produce the protein estimation results of this study. These parameters are only suitable for the genetically modified *E. coli* BL21 (DE3) pET28a cell strain investigated in this study and is mentioned in the Materials and Methods Section. Equation (24) mainly describes the recurrent procedure of offline estimation. Protein estimates were determined from previous protein estimates and offline biomass measurements. First, parameters $k_{m0}$ and $k_{m1}$ were used for determination of $P_{\max,j}$ in Equation (21). This equation also used an approximate value of SGR, $\mu_{j}≅\left(X_{j}-X_{j-1}\right)/\left(X_{j}\cdot ∆t_{j,j-1}\right)$. Equation (24) was only dependent on offline biomass observations in this study, or online biomass estimates in future applications. After calculating the protein value using Equation (24), the “normalized” protein value $P_{x,j}=P_{j}/X_{j}$ served as input for the estimation of the next target protein value by Equation (24). In this way, model fitting used the equation in a recursive manner and had no dependency on target product related state variables.

Protein and biomass model fitting results are presented in Table 3 using the best-fit configurations of models parameters.

Therefore, the average MAE of biomass model fitting since the start of the bioprocess of inoculation is 0.679 g/L and that of product model fitting is 0.246 g/L. The overall average MAPE of biomass model fitting since the start of inoculation is 6.92% and that of product model fitting is 19.87%. The overall average RMSE of biomass model fitting since the start of inoculation is 5.07 g and that of product model fitting is 1.517 g. The MAPE, MAE, and RMSE of the product model fitting neglects the very first measurement after induction, since it has less meaning for MAPE when product synthesis starts. To validate the identified model parameters shown in Table 2, data from six cultivation experiments of the same cell culture were processed.

According to the validation data shown in Table 4, the average MAE of biomass since the start of inoculation is 0.636 g/L and that of product is 0.099 g/L. The overall average MAPE of biomass since the start of inoculation is 7.09% and that of product is 8.22%. The overall average RMSE of biomass since the start of inoculation is 4.577% and that of product is 0.656%.

**Figure 3:**
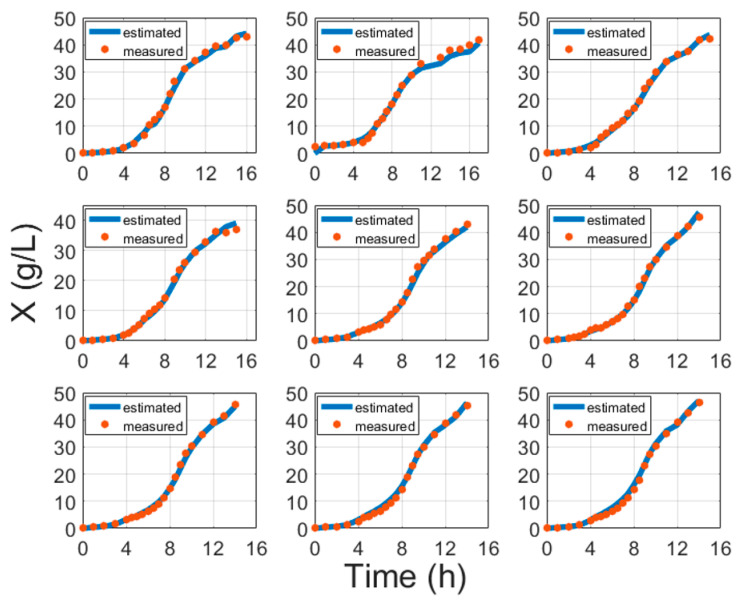
Biomass model fitting results with cultivation processes data, where time is the cultivation time since inoculation in the bioreactor.

**Figure 4:**
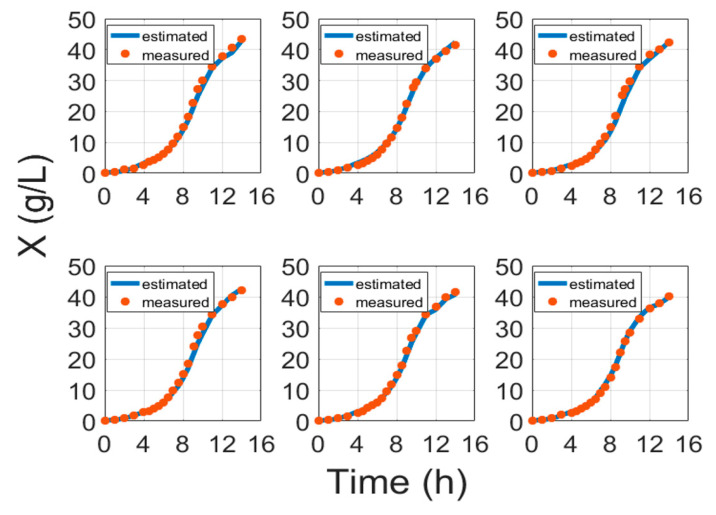
Biomass validation results with cultivation processes data, where time is the cultivation time since inoculation in the bioreactor.

Figure 3 portrays some typical biomass model fitting results and Figure 4 shows biomass validation results. These results show that estimation approaches for biomass concentration and product attained acceptable precision without compromising the simplicity of implementation. The proposed models show a simplistic structure while being accurate and a basis of fundamental knowledge. The main purpose of this paper is to show evidence that biomass and protein model fitting can be handled from the fundamental point of view based on stoichiometry Equation (1) and protein synthesis Equation (19), without the need for an artificial neural network (ANN) or other hybrid black box systems requiring data training [6,41,42,43]. Training procedures normally require huge amounts of training data, while this study proposes an approach that helps with the identification of the parameters once per strain. For comparison, the results of ANN and the model in this paper are compared in Table 5.

**Figure 5:**
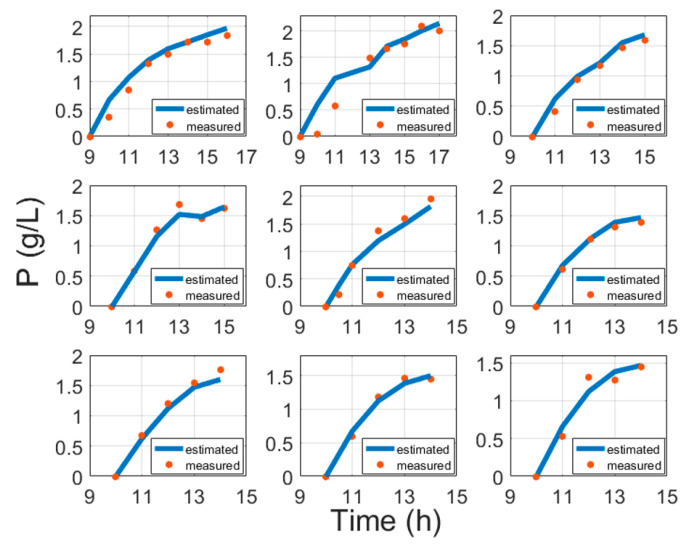
Protein model fitting results compared with cultivation experiment data, where time is the cultivation time since inoculation in the bioreactor.

**Figure 6:**
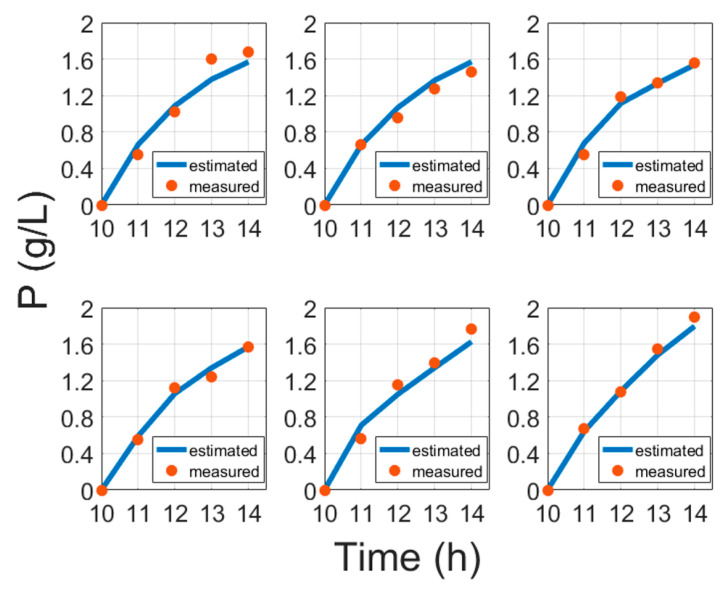
Protein validation results compared with cultivation experiment data, where time is the cultivation time since inoculation in the bioreactor.

Moreover, instead of induction time [6], this study suggests using biomass concentration at induction, which better confirms conventional bioprocess development practices. The results of protein model fitting are shown in Figure 5 and are validated in Figure 6.

## 6. Conclusions

This paper suggests two functional models for biomass and product concentration, which are crucial for the later development of online product and biomass estimators. The biomass model fitting approach uses the stoichiometry model proposed by Luedeking and Piret in 1959. This study assumed that the estimation routines are dependent on stoichiometry parameters of the strain and the biomass concentration at the time of induction. The proposed model fitting method utilizes only few inputs: Specific biomass growth rate and biomass concentration at time of induction. The approach is thus based on fundamental knowledge about biosynthesis. Analysis of process data from 19 cultivation experiments validated the routines. Evaluation errors confirmed that the approach is relevant for model fitting of the *Escherichia coli* BL21 (DE3) pET28a cell strain. The overall average MAE of biomass model fitting was 0.679 g/L and that of product model fitting was 0.246 g/L. The overall average MAPE of biomass model fitting was 6.92% and that of product model fitting was 19.87%. The suggested approach does not depend on any numeric initial optimization conditions and does not require any bioreactor parameters. The proposed approach has certain benefits compared to artificial neural networks. Training procedures normally require a huge amount of training data, while this study proposes an approach that helps with the identification (training) of parameters once per strain. This study suggests using a more general biomass concentration at induction, normally defined in contract or biotechnological protocols.

## Figures and Tables

**Table 1:** Analysis results of biomass concentration models. MAE, mean absolute error; MAPE, mean absolute percentage error.

Model	α	$k_{\beta 0}$	$k_{\beta 1}$	$k_{\beta 2}$	$k_{\gamma }$	MAE	MAPE
Equation (3)	0.996	0.07	0.00084	0	—	1.422	8.85%
Equation (12)	0.997	0	0	0	2.705	0.68	6.92%

**Table 2:** Values of protein model parameters according to Equation (24).

*E. Coli* BL21 (DE3) pET28a
$k_{m0}=0.2346$
$k_{m1}=-0.0172$
$k_{t}=0.0687$

**Table 3:** Analysis of biomass and product concentration models. RMSE, root mean square error.

	Dry Biomass Concentration (Dry Cell Weight, DCW)			Product		
No.	MAE (g/L)	MAPE (%)	RMSE (g)	MAE (g/L)	MAPE (%)	RMSE (g)
1	0.728	6.802	5.212	0.139	5.378	0.571
2	0.762	4.997	6.621	0.231	6.095	0.647
3	0.860	11.022	6.172	0.473	52.526	2.91
4	0.388	4.458	3.085	0.184	13.265	1.248
5	0.798	8.02	6.107	0.527	82.075	3.258
6	0.512	8.82	3.703	0.113	6.7898	0.608
7	0.595	4.787	4.605	0.127	6.957	0.84
8	0.311	4.433	2.191	0.629	35.36	3.757
9	0.576	6.046	4.266	0.178	11.250	1.471
10	0.873	9.017	6.166	0.634	33.844	4.147
11	0.582	5.248	4.468	0.1407	8.286	0.872
12	0.61	5.884	5.264	0.31	19.407	1.946
13	0.7642	5.477	4.962	0.318	39.614	1.834
14	0.404	3.862	3.563	0.056	7.001	0.594
15	0.531	5.724	3.726	0.137	9.681	0.914
16	0.628	7.532	4.503	0.066	4.504	0.401
17	0.86	7.057	6.685	0.16	17.13	1.042
18	1.262	11.767	9.218	0.134	10.328	1.026
19	0.862	10.582	5.933	0.111	8.15	0.738

**Table 4:** Model validation results.

	Dry Biomass Concentration (DCW)			Product		
No.	MAE (g/L)	MAPE (%)	RMSE (g)	MAE (g/L)	MAPE (%)	RMSE (g)
1	0.769	8.594	5.279	0.128	11.947	0.7222
2	0.481	7.39	2.916	0.0813	6.565	0.491
3	0.843	8.107	6.354	0.0563	7.86	0.397
4	0.727	5.25	5.975	0.05	4.996	0.323
5	0.596	7.199	4.17	0.134	8.715	0.821
6	0.402	6.033	2.768	0.149	9.26	1.185

**Table 5:** Comparison of prediction quality of the model in this paper and Gnoth et al. [6] model.

	RMSE (g)		
	Total Biomass	Total Soluble Protein	Total Insoluble Protein
Conventional model from Gnoth et al. [6]	10.81	1.78	0.87
Hybrid network from Gnoth et al. [6]	4.71	1.28	0.62
Model in this study	4.577	-	0.656
